# Supplementary material for: Aspergillus nidulans AmyG Functions as an Intracellular α-Amylase to Promote α-Glucan Synthesis
Source: Microbiol Spectr. 2021 Nov 10;9(3):e00644-21. doi: 10.1128/Spectrum.00644-21 (PMC8592254; doi:10.1128/Spectrum.00644-21)
Supplement: SUPPLEMENTAL FILE 1 — Supplemental material. Download spectrum00644-21_supp_1_seq7.pdf, PDF file, 0.4 MB [file spectrum00644-21_supp_1_seq7.pdf]

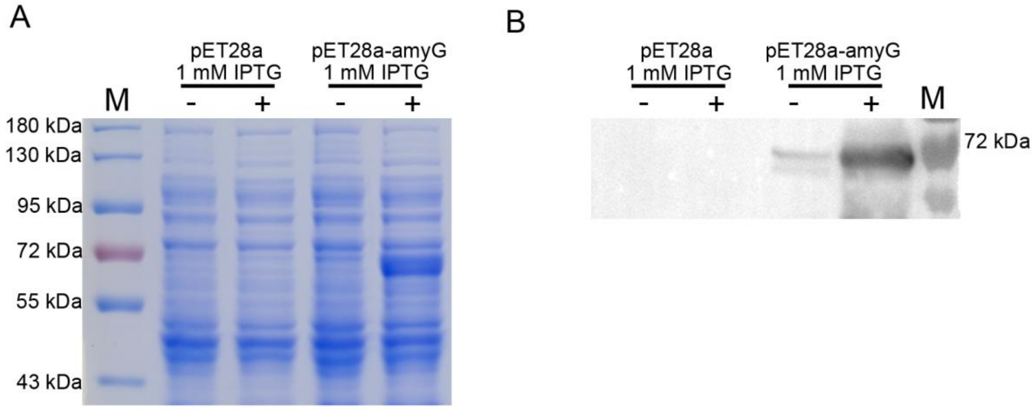

Figure S1 The specificity of *E.coli* expressed AmyG. (**A**) SDS-PAGE analysis of the cell lysate from amyG expressing and non-amyG expressing *E. coli* cells. Cells were harvested before (-) and after (+) IPTG induction. (**B**) Western-blot analysis of AmyG expression. Indicated samples were blotted with an anti-his antibody (AE003, ABclonal, China).

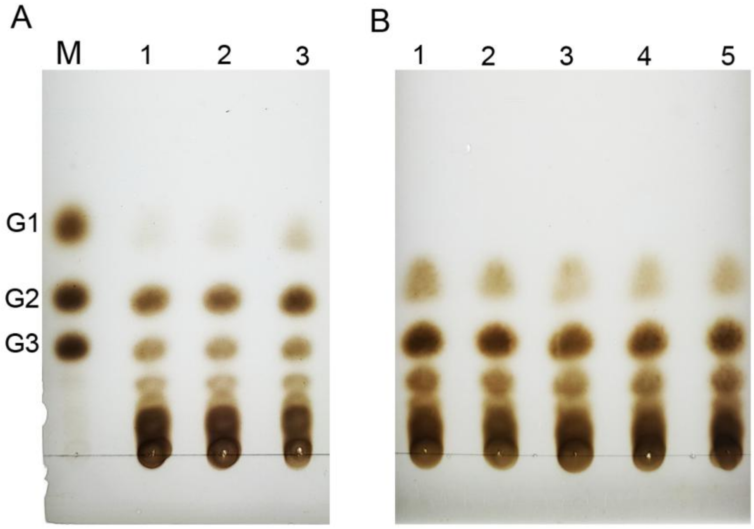

Figure S2 Temperature or PH did not significantly affect AmyG hydrolysis ability. (A) The hydrolysis reactions were set up by incubating starch with purified AmyG for 3 h at different temperature 20 °C (sample 1), 28 °C (sample 2), 37 °C (sample 3). Glucose (G1), maltose (G2), and maltotriose (G3) were loaded as markers.

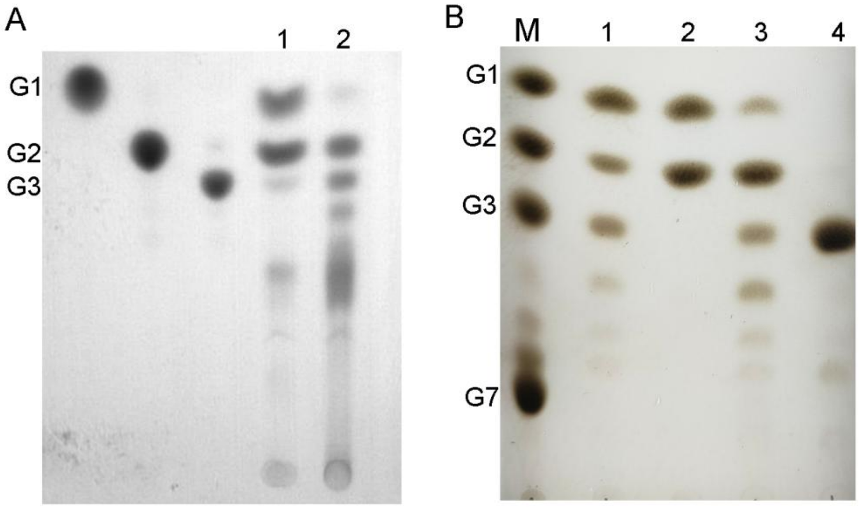

Figure S3 **TLC analyses of reaction products AmyG from hydrolysis assay.** (A) The hydrolysis reactions were set up by incubating starch with commercial α-amylase (sample 1) or purified AmyG (sample 2) for 3 h at 37 °C. Glucose (G1), maltose (G2), and maltotriose (G3) were loaded as markers. (B) The hydrolysis reactions were set up by incubating glucose+maltotriose (sample 1) and glucose+maltose (sample 2) for 3 h at 37 °C. Maltotriose (sample 3) with AmyG or Maltotriose (sample 4) with the cell lysate of non-AmyG expressing *E. coli* cells were incubated for 12 h at 37 °C. Glucose (G1), maltose (G2), maltotriose (G3) and maltoheptaose (G7) were loaded as markers.

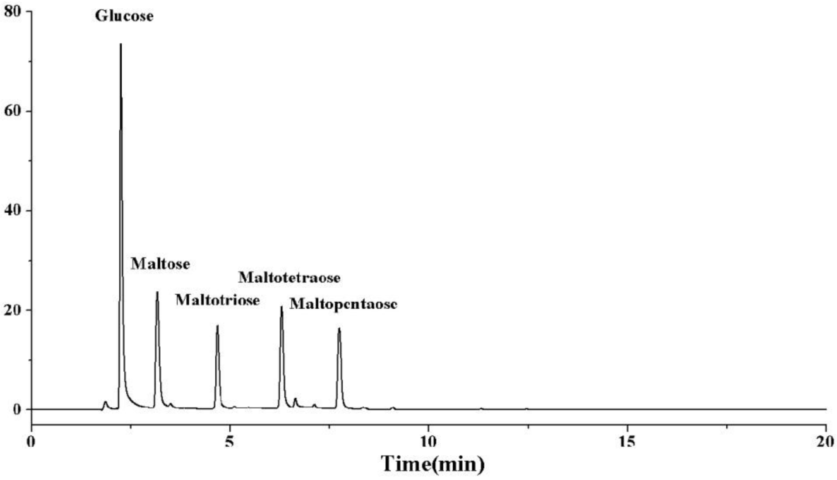

Figure S4 **High performance anion exchange chromatography analyses of standard chemicals.** Standard chemicals were indicated at the top of each peak, G1 glucose, G2 maltose, G3 maltotriose, G4 maltotetraose, G5 maltopentaose, G6 maltohexaose.

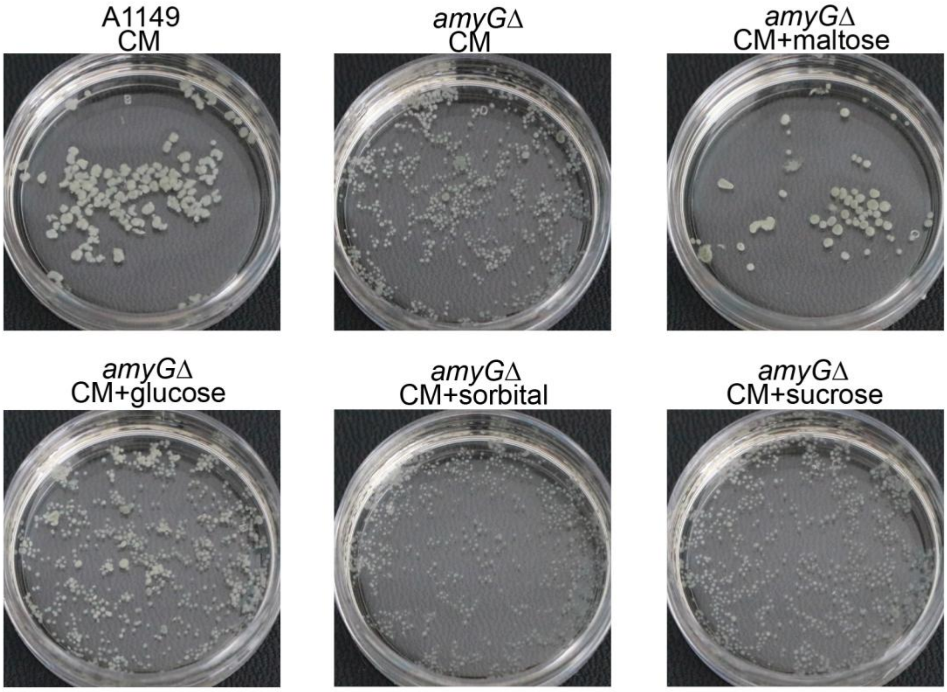

Figure S5 **Osmotic balance cannot rescue the phenotypic defect of *amyG*Δ in shaken liquid medium.** Spores from A1149, and *amyG*Δ strains were inoculated in the indicated media for 24h at 150 r.p.m. and 28 °C. Typical colonies from each medium were transferred to a petri dish for imaging.
